# Is it possible to use the HCR-20 V2 to assess the risk of violent recidivism of French offenders?

**DOI:** 10.1080/20961790.2022.2046370

**Published:** 2022-11-04

**Authors:** Ingrid Bertsch, Robert Courtois, Elodie Gallard, Christian Réveillère, Thierry H. Pham

**Affiliations:** a Department of Psychology, EE 1901 Qualipsy (Qualité de vie et santé psychologique) University of Tours, Tours, France;; b Centre Ressource pour les Intervenants auprès des Auteurs de Violences Sexuelles Centre Val-de-Loire, CHRU de Tours, Tours, France; c Centre de Recherche en Défense Sociale, CRDS, Tournai, Belgium; d Detention Center of Mauzac, Mauzac-et-Grand-Castang, France; e Department of Forensic Psychology, University of Mons-Hainaut, Mons, Belgium

**Keywords:** HCR-20, assessment, recidivism risk, violence

## Abstract

The Historical-Clinical-Risk Management Scale 20 (HCR-20) is a structured tool to assess the risk of violence and assist in its management. French professionals are reluctant to use it because only a few studies have shown its psychometric qualities with French samples. The objective of this study is to test the psychometric qualities of the HCR-20 with samples of violent detainees in France. The HCR-20 and Level of Service/Case Management Inventory (LS/CMI) were administered to 128 violent offenders with an average age of (44.16±12.30) years. We evaluated the reliability, internal consistency and validity of the HCR-20 and conducted an exploratory factor analysis. The results show that the HCR-20 has good psychometric qualities with a sample of French prisoners. Only the Risk domain presents weak results due to the data collection locations and the participants’ age. Correlations were observed between certain factors. The exploratory factor analysis shows four factors explaining 44% of the variance. The continuation of this work will enable French professionals to use sound tools to assess the risk of recidivism.

## Introduction

Advances in the assessment of the risk of recidivism of violent offenders have led to the increasingly systematic use of structured scales [1]. There have been several generations of scales. First, actuarial scales, is a statistical method that assigns points to factors providing a numerical score of the risk of recidivism [2]. These scales are based on static factors, based on past elements, and stable over time. Actuarial scales provide objective, mechanical and reproducible measures of recidivism risk [2]. These scales have the advantage of: (i) provide a more accurate rating of recidivism risk [3] through explicit scoring rules and the support of empirically related risk factors, Level of Service/Case Management Inventory (ii) allow for a numerical estimate of recidivism risk to be compared to reference categories, (iii) does not involve the opinion of the assessor in order to avoid bias, and is simple and quick to use (case-based scoring is possible) [3,4]. However, these scales are often criticized for being too rigid and for not allowing the evolution of a patient to be assessed [5], especially after therapeutic treatment. The second generation, integrated professional judgement provides a risk assessment and an intervention plan [6]. They have the advantage of offering real help in the construction of an intervention plan adapted to the management of the risk of reoffending [7]. The third generation, Structured Professional Judgment models (SPJ), in which the assessor weights risk factors based on his/her observations [8]. They offer flexibility to evaluators by allowing them to weight the rating of variables according to their observations. But, they require clinical knowledge, knowledge of risk factors and interviewing skills [9]. They are also often reserved for psychologists and psychiatrists [10]. But, integrated professional judgement like actuarial valuation, does not provide for the consideration of a weighting of risk factors by the evaluator and therefore does not allow for special circumstances to be considered [11]. The second and third generation scales are based on dynamic risk factors, i.e. reversible factors, allowing the evolution of the offender to be assessed [12].

The Historical-Clinical-Risk Management Scale 20 - version 2 (HCR-20 V2; [13]) is one of the most widely used SPJ scales [14]. Its purpose is to enable systematic assessment of violence risk and to assist in the management of this risk in different situations (pre- or post-sentence) [15]. It makes it possible to assess the risk over a certain period (from several weeks to a few years). The HCR-20 can be used with psychiatric [16], correctional and forensic populations [17,18]. Version 1 [19], version 2 [13] and version 3 [20] of the HCR-20 are composed of 20 items divided into three subscales: (i) Historical items (e.g. previous violence, psychopathy), (ii) Clinical items (e.g. poor introspection, impulsivity), and (iii) Risk management items (e.g. stress problem, treatment noncompliance). These items cover risk factors that are either static (stable over time, historical items) or dynamic (evolving, clinical and risk management items) [21]. The HCR-20 is used in clinical practice and scientific research. Clinically, it aims to provide a qualitative rather than quantitative rating of the risk of recidivism [22]. However, for research purposes, it is possible to apply a rating to each item [23]. Version 2 of the HCR-20 is still widely used in research, with more than 150 clinical data collections in more than 35 countries [20].

The HCR-20 helps clinicians to structure risk assessment and communicate clear and relevant conclusions and guide the decision-making process. Its predictive capacity is based on (i) summing the many relevant risk factors (recognized by data in the scientific literature), (ii) the assessor’s opinion of the weight of these factors, and (iii) the expected nature and intensity of intervention or management strategies needed to mitigate the risk [24]. Unlike actuarial scales, decision-making is not based on a purely numerical algorithm or assessment, to ensure that the score is not based on a single sample, does not omit important risk factors, and has inter-sample validity [20]. Professionals are advised to train in the use of this scale.

The French version of the HCR-20 V2 was translated by T.H. Pham with the collaboration of the Philippe Pinel Institute and validated by Claix and Pham [23] with a sample of 86 patients in a Belgian high-security psychiatric establishment (Internment law^1^). This study revealed good inter-rater reliability (Pearson correlations *r* = 0.73, *P* < 0.05 for the Total score; *r* = 0.85, *P* < 0.01 for the Historical subscale; *r* = 0.65, *P* < 0.05 for the Clinical subscale; *r* = 0.64, *P* < 0.05 for the Risk subscale). Internal consistency was good for the Total score (*α* = 0.74) but lower for the subscales (*r* = 0.61 for the Historical subscale; *r* = 0.47 for the Clinical subscale; *r* = 0.54 for the Risk subscale). Finally, convergent validity, assessed using the Psychopathy Checklist-Revised (PCL-R; [25]), a tool that measures psychopathy and the risk of violent recidivism, showed good correlations for the Total score (Pearson correlations, *r* = 0.63, *P* < 0.01) and the Historical (*r* = 0.60, *P* < 0.01) and Clinical (*r* = 0.55, *P* < 0.01) subscales, but lower correlations for the Risk subscale (*r* = 0.25 *P* < 0.05). The Total score on the HCR-20 was mainly correlated with violent robbery (Pearson correlations, *r* = 0.26, *P* < 0.05), simple and aggravated robberies (*r* = 0.30, *P* < 0.01), assault and battery (*r* = 0.28, *P* < 0.01) and psychotic homicides (*r* = −0.74, *P* < 0.01). In Belgium, after the PCL-R, the HCR-20 is the most widely used tool to assess the risk of recidivism (87.21% of professionals have used it during their career; [9]). In France, the assessment of the risk of recurrence remains mainly assessed with unstructured professional judgment, i.e. without risk scales. To our knowledge, HCR-20 is used very little in France, and rarely in French research [26]. Its use would (i) improve the prediction of risk and therefore the management of the risk of recidivism for offenders and (ii) improve knowledge on recidivism through the use of scales in a research framework. The lack of studies on the psychometric qualities of HCR-20 on French populations is one of the reticence’s expressed by professionals [27].

The aim of this study was to test the psychometric qualities of the HCR-20 in a population of violent prisoners in France. We focused on its internal (i.e. internal validity and reliability) and external (correlations between incidents and the HCR-20) psychometric qualities and conducted an exploratory factor analysis to assess the underlying structure of the scale.

## Method

### Participants

The sample comprised 128 adult male detainees; 86 (67.2%) were sexual offenders (OS) and 42 (32.8%) non-sexual violent offenders (NSVO). Their average age was 44.16 years (SD±12.30, ranges 19–76). They had committed an average of 1.39 offences (SD±0.59; ranges 1–4), including: (i) sexual offences (rape, sexual assault and exhibitionism), (ii) violent offences (homicide, assault and battery, kidnapping/sequestration and arson), and (iii) non-violent offences (simple and aggravated theft, drug offences, possession of weapons, and road traffic offences). They had an average of 10.02 years of education (SD±2.6; ranges 1–15), which corresponds to a *Brevet d’Etudes Professionnelles* (vocational school diploma, 10th grade). They were sentenced to an average of 128 months in prison (SD±84.2; ranges 1–360) and had served an average of 58.9 months at the time of data collection (SD±53.1; ranges 1–384).

### Tools

The HCR-20 [13] is used to predict and manage the risk of violent recidivism. The French version was translated by T.H. Pham with the collaboration of the Philippe Pinel Institute (Quebec, Canada) and validated by [23] with 86 participants. For this study, version 2 of the HCR-20 was used, because the protocol had been developed and the experimenter trained before the development of version 3. [28]. The HCR-20 version 2 assesses three domains, with 20 risk factors: (i) historical (10 factors, e.g. *Previous violence*), (ii) clinical (5 factors, e.g. *Introspection*), and (iii) risk management (5 factors, e.g. *Unrealistic plans*). Each item was scored quantitatively (0 = no evidence, 1 = partial evidence, 2 = definite evidence) in order to obtain a Total score (between 0 to 60) and a score for each subscale [23]. Higher scores indicate greater risk of recurrence. For prison populations, the items on the Risk subscale can be rated from a future perspective (i) within the prison (“in” situation), or (ii) post-discharge (“out” situation). For this study, the “out” situation was used. The HCR-20 has good psychometric qualities [23, 29,30].

The Level of Service/Case Management Inventory (LS/CMI) [31] assesses the general recidivism risk and reintegration capacity of offenders. The French version was translated and validated by Guay [32]. The LS/CMI has 11 sections. Section 1 (the only one used in this study) assesses eight areas, with 43 risk factors: “Criminal history” (e.g. *Has a history of convictions*), “Education and employment” (e.g. *Is currently unemployed*), “Family/marital” (e.g. *Dissatisfaction with marital situation*), “Leisure and recreational activities” (e.g. *Has not recently participated in an organised activity*), “Companions” (e.g. *Has some criminal acquaintances*), “Drug or alcohol problems” (e.g. *Has had problems with alcohol*), “Pro-criminal attitude or orientation” (e.g. *Supportive of crime*), and “Type of antisocial behaviour” (e.g. *Various early antisocial behaviours*). A two-third scoring system yielded a score per domain and a Total score between 0 and 86; higher scores indicate greater risk of recurrence. For this study, only the first part of the LS/CMI was used in this study because it (i) assesses criminogenic risk factors and (ii) quantifies a recidivism risk. The following parts of the LS/CMI allow for the assessment of non-criminogenic factors, which are useful in the general assessment of risk although not directly associated with. As the HCR-20 is only composed of criminogenic factors, it seemed more prudent to use only Section 1 of the LS/CMI. The LS/CMI has good psychometric qualities [32,33].

Several reasons supported the selection of the LS/CMI in order to assess the convergent validity of the HCR-20. The LS/CMI present similarities to the HCR-20 as it assesses general recidivism as well as violent recidivism (in the same way as the HCR-20) [11] and is composed of actuarial and dynamic factors [31]. These two tools are often assessed together for the study of recidivism (for example [34,35]). Finally, the use of the LS/CMI to assess the concurrent validity of the HCR-20 is not uncommon and has been undertaken before [36].

### Procedure

This study was approved by a French centre (Espace de Reflexion Ethique en région Centre (EREC) avis favorable du dossier n°2017 077) for ethics in health care. Data were collected between March 2018 and February 2020. Once the healthcare teams were informed of the goal, the patient inclusion criteria and the study process, they suggested that the evaluators meet with potential participants. After a first meeting with the participant explaining the study, a second meeting allowed them to sign the consent sheet and answer any questions. The inclusion criteria for participants were: (i) to be an adult, (ii) to be currently convicted of having committed acts of violence and (iii) to speak French. The exclusion criteria were: (i) to be over 70 years  old, (ii) to be warned and (iii) to have a psychiatric pathology leading to an inability to express oneself or severe cognitive impairment. First, the main evaluator conducted semi-structured interviews with the participants, designed to enable each risk scale to be scored in line with the tool’s guidelines. The interview had four sections: Section 1 focused on the participant’s childhood and adult criminal history (e.g. types of past offences, description of victims); Section 2 concerned the offences for which the participant had been convicted (e.g. nature of the offence, description of the victim); Section 3 concerned the participant’s psychopathological and medical history (e.g. history of mental illness, alcohol/drug use). Finally, Section 4 concerned elements of the participant’s social history (e.g. education, employment, family relationships). The evaluator then consulted the participants’ medical records in order to complete or modify the knowledge obtained through the semi-structured interviews. The evaluator had been trained to use all these tools with approved trainers (T.H. Pham for HCR-20 and J.P. Guay for LS/CMI). For inter-rater reliability, a double-blind scoring was carried out by a second assessor, also trained in the use of the HCR-20 and LS/CMI scales with approved trainers, from the data collected by the main assessor. The inter-rater reliability assessment was carried out for seven OS participants (8.14%).

### Data analysis

All the results were analyzed using SPSS© IBM© version 25 (Armonk, NY). It should be noted that the item “H7 psychopathy” was omitted in this study. This item could not be rated due to the lack of substantiated information. As for the HCR-20 results, the sample in this study does not follow a normal distribution, so all tests used are non-parametric. The internal consistency was calculated using the Cronbach alpha coefficient. Inter-rater reliability was assessed using Spearman correlations and intra-class correlation indices (ICC). According to Koo and Li [37], two-way mixed-effects model with absolute-agreement and Kappa means measures is the model of choice for our study. Cohen’s *d* coefficients were calculated to analyze the similarities between the data of the current sample and those of a sample of patients placed in a Belgian high-security psychiatric establishment. The links between HCR-20 scores and the types of offence committed were analyzed using (i) Mann-Whitney tests for comparison of averages and (ii) point-biserial correlation coefficients. In view of the large number of correlations carried out, a Bonferroni correction was applied. For this, the alpha score (here *P* = 0.05) was divided by the number of tests (here 4). The significance level of the *P*-value is therefore 0.01 instead of 0.05. Spearman correlations were conducted to understand the relationship between HCR-20 scores and total LS/CMI scores. Finally, an exploratory factor analysis was conducted, checking the Bartlett test and the KMO index. A principal component analysis (PCA) was conducted with the 20 items of the HCR-20 to identify the factor structure. We used: (i) Cattell’s test to determine the number of factors to be retained, and (ii) Varimax rotation to maximize variance. This allowed us to assess an exploratory model of the internal validity of the scale. The items included in each of the factors had an absolute value greater than 0.40. The excluded items had an absolute value lower than 0.40.

## Results

### Descriptive results

Table 1 summarizes the average scores obtained on the HCR-20 and LS/CMI scales. In the HCR-20, participants obtained an average Total score of 14.21 (SD±6.77; ranges 0–28), a Historical subscale score of 7.17 (SD±4.56; 0–17), a Clinical subscale score of 3.19 (SD±2.00; 0–8), and a Risk subscale score of 3.84 (SD±1.92; 0–9). The majority of participants (*n* = 91, 71.1%) belongs to the low risk category whereas no participant belongs to high risk category. The average Total LS/CMI score was 16.76 (SD: 7.21; 3–34). The majority of participants belong to moderate (*n* = 52, 40.6%) and high (*n* = 40, 31.3%) risk categories.

**Table 1.** A verage scores on HCR-20 and LS/CMI. (*N* = 128)

Scale	Score^a^				
	Mean	SD	Median	Min	Max
HCR-20					
Total score	14.21	6.77	14	0	28
Historical	7.17	4.56	6	0	17
Clinical	3.19	2.00	3	0	8
Risk	3.84	1.92	4	0	9
Risk levels					
Low	91 (71.1%)				
Moderate	37 (28.9%)				
High	0 (0)				
LS/CMI					
Total score	16.76	7.21	16	3	34
Criminal history	3.73	1.99	4	0	8
Education and Employment	3.45	2.54	3	0	9
Family/marital	2.22	1.49	2	0	13
Leisure and Recreational Activities	2.85	0.76	3	0	4
Companions	0.77	0.75	1	0	2
Drug or alcohol problems	1.44	0.76	1	0	8
Pro-criminal attitude or orientation	1.22	1.03	1	0	4
Type of antisocial behaviour	1.15	1.09	1	0	4
Risk levels					
Very low	0 (0)				
Low	30 (23.4%)				
Moderate	52 (40.6%)				
High	40 (31.3%)				
Very high	6 (4.7%)				

HCR-20: Historical-Clinical-Risk Management Scale-20; LS/CMI: Level of Service/Case Management Inventory.

^a^Scores of Risk levels are presented as percentage in parentheses.

### Reliability and internal consistency

Table 2 presents the HCR-20 and LS/CMI inter-rater reliability and internal consistency for our sample. The inter-rater reliability for the HCR-20 Total score was excellent (*ρ* = 0.93; *P* < 0.01; ICC = 0.96 for the mean measure) and good for LS/CMI (*ρ* = 0.84, *P* < 0.01; ICC = 0.88). Internal consistency was acceptable for the HCR-20 Total score (*α* = 0.79) It was highest for the Historical factor (*α* = 0.80) and lowest for the Risk factor (*α* = 0.29). Finally, internal consistency was acceptable for LS/CMI Total score (*α* = 0.72).

**Table 2.** Inter-rater reliability and internal consistency of the HCR-20 and LS/CMI.

	Inter-rater reliability		Internal consistency	
	Scale	Spearman’s correlations (*ρ*)		Intra-group index (ICC)	Cronbach alpha (*α*)
HCR-20				
Total score	0.93**		0.96	0.79
Historical	0.99**		0.99	0.80
Clinical	0.73*		0.80	0.48
Risk	0.64*		0.76	0.29
LS/CMI				
Total score	0.84**		0.88	0.72

*P < 0.05; **P < 0.01.

HCR-20: Historical-Clinical-Risk Management Scale-20; LS/CMI: Level of Service/Case Management Inventory.

### Convergent validity

The HCR-20 Total score and Historical subscale score were highly correlated with the LS/CMI Total score (*ρ* = 0.80, *P* < 0.01 and *ρ* = 0.79, *P* < 0.01, respectively). Correlations between the Clinical and Risk subscales and the LS/CMI Total score were low (*ρ* = 0.57, *P* < 0.01 and *ρ* = 0.38, *P* < 0.01, respectively).

### Using the HCR-20

#### Comparisons between French and Belgian data

Table 3 presents comparisons of average scores between the current sample and a sample of psychiatric patients interned in Belgium. Three conventional effect size thresholds were proposed by Cohen [38]: 0.2 (small effect), 0.5 (moderate effect) and 0.8 (large effect). A moderate effect size was observed for the mean age of the French and Belgian samples (respectively, 44.16, SD ± 12.30 *vs.* 36.71, SD ± 16.28; *d* = 0.52; *r* = 0.25). The mean total HCR-20 scores for both samples show a large effect size (respectively, 14.21; SD ± 6.78 *vs.* 23.30; SD ± 6.31; *d* = –1.39; *r* = –0.57).

**Table 3.** Comparison of HCR-20 averages between a French and a Belgian sample.

	French sample (*N* = 128)		Belgian sample (*N* = 202)		
	Mean (SD)		Mean (SD)	Cohen’s *d*	Coefficient *r*
Total score	14.21 (6.78)		23.30 (6.31)	−1.39	−0.57
Historical	7.17 (4.56)		12.40 (3.76)	−1.25	−0.53
Clinical	3.19 (2.00)		4.95 (2.22)	−0.83	−0.38
Risk	3.84 (1.92)		6.02 (1.99)	−1.12	−0.49
Age	44.16 (12.30)		36.71 (16.28)	0.52	0.25

HCR-20: Historical-Clinical-Risk Management Scale-20.

#### Correlations between HCR-20 scores and types of offence committed

Table 4 presents the point-biserial correlations with Bonferroni correction between HCR-20 scores and the offences committed by the participants. The Total score on the HCR-20 was moderately and negatively correlated with sexual assault (*ρ* = –0.34, *P* < 0.01), moderately and positively correlated with assault and battery (*ρ* = 0.25, *P* < 0.01) and with theft (*ρ* = 0.26, *P* < 0.01). These results are confirmed by comparisons of averages between the Total score on the HCR-20 and the offences committed (15.84 ± 6.74 *vs.* 11.00 ± 5.64, *Z* = –3.79, *P* < 0.001 for sexual assault; 13.15 ± 6.55 *vs.* 17.03 ± 6.62, *Z* = –2.82, *P* < 0.005 for assault and battery; 13.81 ± 6.66 *vs.* 22.33 ± 2.25, *Z* = –2.90, *P* < 0.004 for theft).

**Table 4.** Point-biserial correlation coefficients with Bonferroni correction between HCR-20 scores and type of offence.

Type of offence	HCR-20				
	Number of offence	Total score	Historical	Clinical	Risk
Sexual offences					
Rape	60	−0.05	−0.03	0.12	0.03
Sexual assault	43	−0.34**	−0.33**	−0.24**	−0.12
Sexual exhibition	1	0.10	0.08	0.12	0.08
Violent offences					
Homicide	23	−0.07	−0.09	−0.06	−0.03
Assault and battery	35	0.25**	0.26**	0.32**	−0.05
Kidnapping and false imprisonment	5	0.10	0.06	0.16	0.03
Arson	2	0.08	−0.02	0.17	0.12
Non-violent offences					
Simple and aggravated theft	6	0.26**	0.29**	0.14	0.03
Drug offences	3	0.11	0.09	0.14	0.02
Possession of weapons	2	0.08	0.04	0.14	−0.01
Traffic offence	1	−0.14	−0.11	−0.06	−0.15

**P < 0.01.

HCR-20: Historical-Clinical-Risk Management Scale-20.

The Historical and Clinical subscales were moderately and negatively correlated with sexual assault (respectively, *ρ* = –0.33, *P* < 0.01; *ρ* = –0.24, *P* < 0.01) and moderately and positively correlated with assault and battery (respectively, *ρ* = 0.26, *P* < 0.01; *ρ* = 0.32, *P* < 0.01). The Historical subscale was moderately and positively correlated with simple and aggravated theft (*ρ* = 0.29, *P* < 0.01). The Risk subscale showed no correlation with the type of offence.

### Factorial analysis

**Figure 1.**
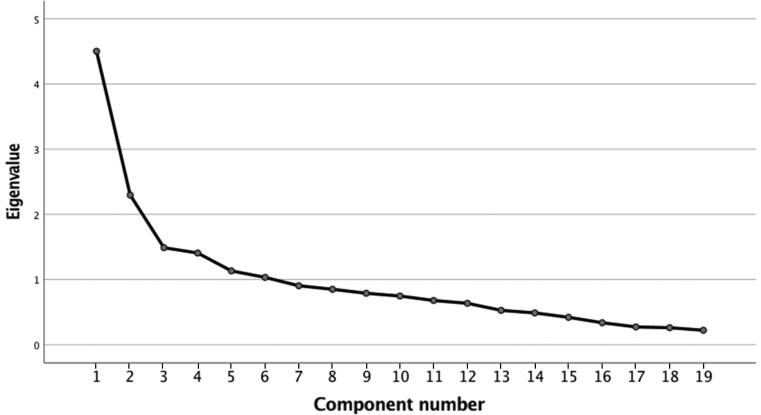
Cattell’s Elbow of the Exploratory Factor Analysis of the HCR-20. HCR-20: Historical-Clinical-Risk Management Scale-20.

The Bartlett (*P* < 0.001) and KMO (0.72) tests justified a factor analysis. Figure 1 and Table 5 show details of the exploratory factor analysis with Varimax rotation performed on the HCR-20. The results of this analysis highlight three subscales, “Historical”, “Clinical”, and “Social”, explaining 44% of the total variance. Cronbach’s alpha coefficients measuring the internal consistency of this model and the subscales were 0.60 for the Total score, 0.83 for the Historical subscale, 0.61 for the Clinical subscale, and 0.45 for the Social subscale.

**Table 5.** Exploratory factor analysis of the HCR-20.

	Rotation of the component matrix		
Factors loading >0.40	“Historical” subscale	“Clinical” subscale	“Social” subscale
Personality disorder (H9)	0.78		
Impulsivity (C4)	0.75		
Previous violence (H1)	0.73		
Early maladjustment (H8)	0.71		
First violence at young age (H2)	0.67		
Prior supervision failure (H10)	0.61		
Employment (H4)	0.60		
Substance abuse (H5)	0.46		
Disease symptoms (C3)		−0.64	
Mental illness (H6)		−0.56	
Stress (R5)		−0.44	
Non-compliance with treatment measures (R4)		0.55	
Introspection (C1)		0.60	
Treatment resistance (C5)		0.62	
Negative attitudes (C2)		0.41	
Destabilizing factors (R2)			0.66
Personal support (R3)			0.66
Intimate relationships (H3)			0.42
Unrealistic plans (R1)	–	–	–
Explained variance (%)	23.70	12.08	7.84
Cronbach’s alpha coefficients	0.83	0.61	0.45

The letters and numbers in brackets correspond to the names of the items. HCR-20: Historical-Clinical-Risk Management Scale-20; H: historical; C: clinical; R: risk.

## Discussion

The aim of this study was to assess the psychometric qualities of the HCR-20 with a French population of violent detainees. The results show satisfactory internal and external validity of the HCR-20. Comparison with a sample of psychiatric patients interned in a Belgian establishment revealed differences in scores. The Total score and the Historical subscale are positively associated with the offences of “simple and aggravated theft”, “assault and battery”, and negatively with “sexual offences”; the Clinical subscale is positively associated with “assault and battery” and negatively with “sexual assault”. Finally, exploratory factor analysis identified three factors corresponding to the subscales (“Historical”, “Clinical” and “Social”), explaining 44% of the total variance with satisfactory internal consistency.

The results of this study indicate that the HCR-20 has good inter-rater reliability and internal consistency apart from the Risk subscale. The inter-rater reliability results are similar to the data using the English version of the HCR-20. A study with a sample of 75 offenders in a Canadian federal prison found a Pearson’s correlation coefficient of 0.80 for the Total score of the HCR-20 [15]. Another study with 250 detained offenders showed an intra-class correlation index of 0.92 for the Total score, 0.92 for the Historical subscale, 0.74 for the Clinical subscale, and 0.70 for the Risk subscale [29]. The Belgian data, using the French version of the HCR-20, showed similar results to those of the French version for (i) Pearson correlations (*r* = 0.73, *P* < 0.05 for the Total score, *r* = 0.85, *P* < 0.01) and (ii) intra-class correlation indices (ICC = 0.82 as a mean measure for the Total score; [23]. The internal consistency was found to be similar to the North American data (*r* = 0.78; [15]) and to the Belgian French-speaking data (*r* = 0.74 for the Total score; [23]). It is also similar to a study of 60 detained offenders in France (*r* = 0.84 for Total score; [39]).

The results show a satisfactory convergent validity of the HCR-20. Positive correlations between the HCR-20 scores and the total LS/CMI score were observed in a sample of 72 psychiatric patients who had committed serious violence (Pearson’s coefficient, *r* = 0.75 for the Total score, *r* = 0.67 for the Historical subscale, *r* = 0.53 for the Clinical subscale, and *r* = 0.48 for the Risk subscale; [36]).

Comparisons between the present French study and the Belgian study show differences between these two populations, overall scores being significantly higher in the Belgian study. This can be explained by the nature and age of the participants; all the Belgian participants were held in a secure psychiatric unit, whereas the French participants were detained in traditional prison establishments. The Belgian system holds patients-offenders (for a minimum of 3 months) presenting with severe dementia or mental disorder and considered a danger to society. It is thus both a security and a treatment measure of indefinite duration [40,41]. The participants in the Belgian sample thus come from an “at-risk” population, and the majority had already served one or more prison sentences. Participants in the French sample, by contrast, were in institutions where the focus is on the length of the sentence and not the risk. The Belgian participants were also younger. Age is itself a significant risk factor for recidivism [42] and influences the severity [43] and the intensity [44] of the risk, which decrease with age [43,44]. The score on the Clinical subscale is particularly low for the French population. The Clinical subscale is composed of current factors of psychiatric or psychological disorders. However, the French sample comes from a care unit that offers regular psychological care, which may explain the low presence of these clinical factors. In addition, there may have been a bias in the choice of participants by the caregivers (who may have excluded patients who were too ill).

The Total score on the HCR-20 was positively correlated with “assault and battery” and “theft”, and negatively correlated with “sexual assault”. No correlation was observed with “homicide”. These results are consistent with findings obtained with psychiatric patients interned in Belgium [23] and prisoners in France [39]. Although consistent with the study by Pham et al. [39], the negative correlation between HCR-20 and sexual aggression might seem surprising. The population of this study is mainly composed of sexual offenders of children, which present less antisocial profiles [45], an important factor in assessing the risk of violent recidivism. Sexual offenders of children are mainly concerned with sexual recidivism [46], which has factors in common with the risk of violent recidivism, but also has its own factors, not present in the HCR-20. Other scales are more specialized in assessing the risk of sexual recidivism [47]. A higher correlation of the HCR-20 with violent offences is not surprising in view of the test’s objective (to assess the risk of violent behaviour) and the importance of historical and antisocial items [39].

In our study, as in the one by Pham et al. [39], we observed differences in reliability and internal consistency between the three subscales of the HCR-20. The Historical subscale has the highest internal reliability and consistency, while the Risk subscale has the lowest. Internal consistency as measured by Cronbach’s alpha has the disadvantage of being sensitive to the number of items [48,49]. There are fewer items in the Risk subscale than in the Historical subscale (10 *vs.* 5 items). The more the items are based on past (factual) and objective data (collected from official documents), the greater the reliability and consistency. This is the case for the Historical subscale. By contrast, the more the items are based on future, subjective (collected using the respondent’s own words) and disparate data, the weaker the reliability and consistency. This is the case for the Risk subscale [23,48]. Finally, at the time of conducting this study, the participants had served an average of 46% of their custodial sentence. At this stage of their sentence, it is difficult to anticipate a life plan after release (finding a job, housing, etc.). However, the Risk subscale was rated from a post-prison perspective (“out” situation), making its prediction more projective and less certain.

The exploratory factor analysis proposed in this study is satisfactory with acceptable internal consistency. It revealed three subscales, with good internal consistency and explaining 44% of the variance. The History subscale (8 items) explained 23.70% of the variance and had the best internal consistency (*r* = 0.83); it is mainly composed of historical items and covers almost all the items of the initial HCR-20 Historical subscale. The “Clinical” subscale (7 items) explained 12.08% of the variance and had acceptable internal consistency (*r* = 0.61); it is mainly composed of items from the Clinical and Risk subscales of the initial version of the HCR-20. Finally, the “Social” subscale (3 items) explained 7.84% of the variance and had a lower internal consistency (*r* = 0.45); it is mainly composed of items from the Risk subscale of the initial version of the HCR-20. Again, the sensitivity of Cronbach’s alpha to the number of items may explain the low internal consistency of this subscale [48,49]. For the Historical and Clinical subscales, the factor analysis shows that internal consistency was higher in our sample than in the Belgian sample (respectively, *r* = 0.61 *vs.* *r* = 0.84 for the Historical subscale; *r* = 0.47 *vs.* *r* = 0.60 for the Clinical subscale; *r* = 0.54 *vs.* *r* = 0.45; [23]).

This study has a number of limitations. It was carried out in the specific context of detention, which is not a situation of major risk of violence. In principle, the rating of the HCR-20 and LS/CMI scales is optimal when multiple sources of substantiated information are available (participant’s account, medical record, criminal record, prison report, etc.; [13,50]). In this study, scoring was more difficult for a sub-group of participants (*n* = 85, 66.41%) whose file contents were heterogeneous in terms of (i) quantity of information (some files only contained current information while others provided details of the offender’s past), and (ii) quality of information (some files only contained medical information while others contained criminal records). Due to the short time scale of the study, no followup data could not be collected, blocking possibility to assess predictive validity. Finally, the size of our sample (*n* = 128) was relatively small in relation to the normal standards for factor analysis. Caution should thus be exercised when generalizing the results to the entire prison population.

Despite these limitations, this study made it possible (i) to assess the psychometric qualities of the HCR-20 with a sample of violent detainees in France, (ii) to observe a congruence of these qualities with data in the international literature, and (iii) serious viole to propose a new factor model. In France, the use of risk scales is highly controversial despite evidence from case studies showing the clinical applicability of the HCR-20 [51]. To develop their use, professionals must be able to rely on instruments that have been validated with French offenders. The HCR-20 has demonstrated its qualities for assessing the risk of violent recidivism, and seems appropriate to include a more global assessment model targeting specific populations (e.g. sexual offenders) and all types of risk (violent, sexual and general). Future studies could focus on validating these results with a larger sample of detainees, and with samples of psychiatric patients in general and those with higher levels of risk (e.g. those interned in secure units). Thus, the psychometric qualities of the HCR-20 will be validated on a variety of French patient populations. They could also continue to investigate this new factor model to guarantee the reliability of the HCR-20 structure, and test the predictive validity of the HCR-20 by following up participants over a period of more than 4 years. Futures studies will enable French professionals to be sure of the possible use of HCR-20 with their patients.

## Footnotes

1 A law allowing the indefinite internment of offenders with a severe mental disorder that makes them unable to control their actions [52].
